# Correlation Analysis for Protein Evolutionary Family Based on Amino Acid Position Mutations and Application in PDZ Domain

**DOI:** 10.1371/journal.pone.0013207

**Published:** 2010-10-06

**Authors:** Qi-Shi Du, Cheng-Hua Wang, Si-Ming Liao, Ri-Bo Huang

**Affiliations:** 1 State Key Laboratory of Bioenergy Enzyme Technology, National Engineering Research Center for Non-food Biorefinery, Guangxi Academy of Sciences, Nanning, Guangxi, China; 2 Life Science and Biotechnology College, Guangxi University, Nanning, Guangxi, China; 3 Biotechnology and Pharmaceutical Engineering, Nanjing University of Technology, Nanjing, Jiangsu, China; 4 Gordon Life Science Institute, San Diego, California, United States of America; Aarhus University, Denmark

## Abstract

**Background:**

It has been widely recognized that the mutations at specific directions are caused by the functional constraints in protein family and the directional mutations at certain positions control the evolutionary direction of the protein family. The mutations at different positions, even distantly separated, are mutually coupled and form an evolutionary network. Finding the controlling mutative positions and the mutative network among residues are firstly important for protein rational design and enzyme engineering.

**Methodology:**

A computational approach, namely amino acid position conservation-mutation correlation analysis (CMCA), is developed to predict mutually mutative positions and find the evolutionary network in protein family. The amino acid position mutative function, which is the foundational equation of CMCA measuring the mutation of a residue at a position, is derived from the MSA (multiple structure alignment) database of protein evolutionary family. Then the position conservation correlation matrix and position mutation correlation matrix is constructed from the amino acid position mutative equation. Unlike traditional SCA (statistical coupling analysis) approach, which is based on the statistical analysis of position conservations, the CMCA focuses on the correlation analysis of position mutations.

**Conclusions:**

As an example the CMCA approach is used to study the PDZ domain of protein family, and the results well illustrate the distantly allosteric mechanism in PDZ protein family, and find the functional mutative network among residues. We expect that the CMCA approach may find applications in protein engineering study, and suggest new strategy to improve bioactivities and physicochemical properties of enzymes.

## Introduction

Coevolution is a well known phenomenon in biological world. However, coevolution in families of proteins and genes is still an open topic [1]. Conservation and mutation are two opposite aspects of functional evolution of protein family. It is commonly accepted that the evolution of a protein family is the result of large-scale random mutagenesis, with selection constraints imposed by their biological functions. In the studies of statistical analysis for protein evolutionary family the following two basic hypotheses were recognized widely, which were derived from the empirical observation of sequence evolution [2]. (i) The lack of evolutionary constraint at one position should cause the distribution of observed amino acids at that position in the MSA (multiple structure alignment) to approach their mean abundance in all proteins, and deviances from the mean values should quantitatively represent conservation. (ii) The functional coupling of two positions, even if distantly positioned in the structure, should mutually constrain evolution at the two positions, and these should be represented in the statistical coupling of the underlying amino acid distributions [3], [4], [5].

The protein functions are not only determined by the interactions between local residues, but also depend on nonlocal, long-range communication between amino acids [6]. For example, information transmission between distant functional surfaces on signaling proteins [7], the distributed dynamics of amino acids involved in enzyme catalysis [8], [9], [10], and allosteric regulation in various proteins [3], [11] all represent manifestations of nonlocal interactions between residues. To the extent that these features contribute to defining biological properties of protein lineages, it is expected that the underlying mechanisms represent a long-range mutative network, consisting of local and nonlocal residues. Understanding the fundamental basis of long-range communication represents a major challenge in structural biology, which is significantly important for enzyme engineering and rational protein design.

Over the last ten years, a considerable methodological effort has been made to detect coevolution in protein and gene families at molecular level. In the method developed by Dunn et al. [12], [13] information theory was used to reduce the random noise in the identification of coevolving positions. Dutheil and Galtier reviewed the literatures about molecular coevolution between or within residues in gene and protein families [1], [14]. A successful approach, namely statistical coupling analysis (SCA), was developed by Ranganathan's group [2], [15], [16], [17], which focused on the conservations between coupling positions (sectors). However, in the enzyme engineering [18] what we are more interested is that how the amino acid mutations at certain positions modify (or improve) the biological functions (including bioactivity, thermostability, pH tolerance, and other properties) of enzymes. In the rational protein design we want to know the dominative positions for functional evolution and the mutually mutative network among positions in three dimensional structures of proteins.

In this study a computational approach, namely amino acid position conservation-mutation correlation analysis (CMCA), is developed to predict mutually mutative positions and find the evolutionary network in protein family. Unlike traditional SCA (statistical coupling analysis) approach [2], [15], [16], [17], which is based on the statistical analysis of position conservations, the CMCA focuses on the correlations of position mutations in a protein evolutionary family. We expect that the CMCA approach may find applications to rational protein design and enzyme engineering to improve bioactivities and physicochemical properties of enzymes.

## Results

**Figure 1:**
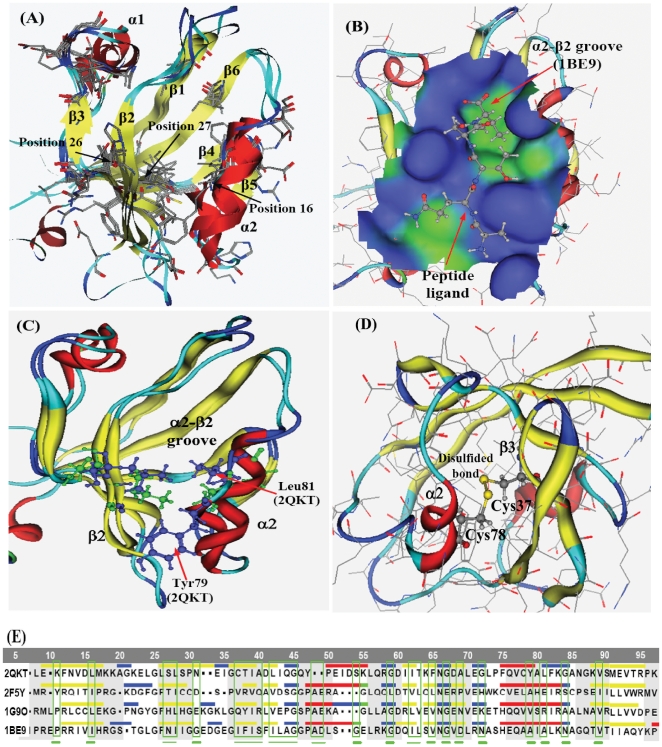
Structure alignment of four PDZ proteins and results of position conservation-mutation correlation analysis. (**A**) The 3D structural alignment of 2QKT, 2F5Y, 1G9O, and 1BE9. The 27 residues, at the positions having higher mutation correlation coefficients (see Table 1), are shown in stick render. (**B**) The surface of α2-β2 groove of 1BE9 and the peptide ligand in 1BE9. Blue is for hydrophilic surface and green is for hydrophobic surface. (**C**) The residues at the controlling positions for ligand affinity. The sizes of Tyr79 and Leu81 of 2QKT (blue) are much bigger than the Ala76 and Ala78 of 1BE9 (green). (**D**) The disulfide bond between Cys37 and Cys78 of 2QKT. (**E**) Sequence alignment of four PDZ proteins (2QKT, 2F5Y, 1G9O, and 1BE9). The residues, at the positions having high position mutation correlation coefficients (see Table 1), are indicated by green frames.

In this study the PDZ domain family is selected as a model system to demonstrate the CMCA approach. The PDZ domain is a common structural domain found in the signaling proteins [19] of bacteria, yeast, plants, viruses [20], animals [21], [22], and human [23], [24]. PDZ domains consist of 90–100 amino acid modules that adopt a six-stranded β sandwich configuration with two flanking α helices (Fig. 1). Target C-terminal ligands bind in a surface groove formed between the β2 strand and α2 helix and make a number of interactions that determine both general and sequence specific recognition [25], [26], [27]. Both the overall three-dimensional structure and most details of ligand recognition are highly conserved in the PDZ family despite considerable sequence divergence [28]. PDZ domains well represent protein binding motifs for which four high-resolution structures of distantly related members exist [25], [29], [30]. These domains help anchor transmembrane proteins to the cytoskeleton and hold together signaling complexes [31], [32].

In this study we use a multiple structure aligned PDZ database [33] consisting of 240 PDZ proteins. After sequence alignment there are 129 positions in the PDZ database, and after deletion of the unnecessary gaps, 27 positions are deleted and the reduced database contains 102 positions.

### Conservation correlation analysis of PDZ

**Figure 2:**
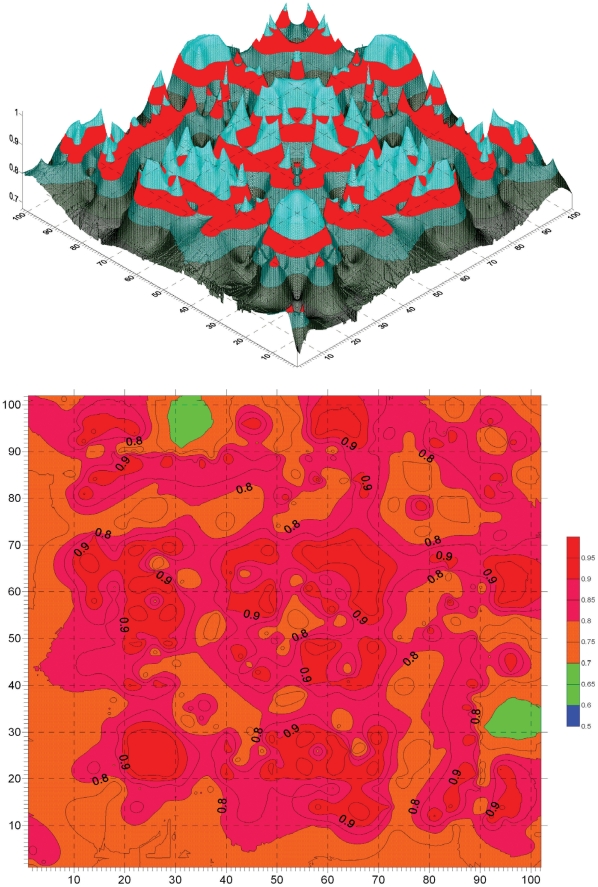
Graphical representation of position conservation correlation matrix R^(con)^_L×L_ of PDZ. (**A**) The relief map of position conservation correlation matrix R^(con)^_L×L_ of PDZ database. The red bands indicate the region with correlation coefficients between 0.80 to 0.85. The relief map of R^(con)^_L×L_ is complementary to the relief map of position mutation correlation matrix R^(mut)^_L×L_ (Fig. 3). (**B**) The contour map of position conservation correlation matrix R^(con)^_L×L_ of PDZ database. For a clear view the matrix elements *r* ^(con)^ *_i,j_* less than 0.5 are filtered, and the elements (*r* ^(con)^ *_i,i_* = 1) on diagonal line are not shown. In the primer PDZ database the sequence length is 129, including gaps, which are inserted in the multiple alignment. After deletion of the unnecessary gaps, the length is reduced to L = 102. The position conservation correlation matrix R^(con)^_L×L_ is symmetric to the diagonal line. The map is colored according to the values: the regions with value higher than 0.90 are colored in red, higher than 0.80 in pink, and higher than 0.70 in orange.

Following the procedure described in Method section, we first perform the conservation position correlation analysis to the PDZ protein family. The position conservation correlation matrix R^(con)^_L×L_ of PDZ is graphically shown in Fig. 2. The matrix R^(con)^_L×L_ is symmetric to the diagonal line. For a clear view the matrix elements *r* ^(con)^ *_i,j_* less than 0.5 are filtered. The matrix elements on the diagonal line, whose values are 1 (*r* ^(con)^ *_i,i_* = 1), are not shown, because the diagonal elements are self correlation coefficients, having no statistical meaning. Fig. 2 A is the relief map of position conservation correlation matrix R^(con)^_L×L_ of PDZ database. The red bands indicate the region with correlation coefficients from 0.80 to 0.85. Fig. 2 B is the contour map of position conservation correlation matrix R^(con)^_L×L_ of PDZ database. The map is colored according to the values: the regions with value higher than 0.90 are colored in red, higher than 0.80 in pink, and higher than 0.70 in orange.

In Fig. 2 A most places are high peaks and in Fig. 2 B most areas are in red color, meaning that in many sequence positions the residues are highly conserved, consistent to the high conservation of PDZ family. It is difficult to dig out detailed information from the position conservation correlation matrix R^(con)^_L×L_ of PDZ database, because too many conservative positions complicate the analysis.

### Mutation correlation analysis of PDZ

**Figure 3:**
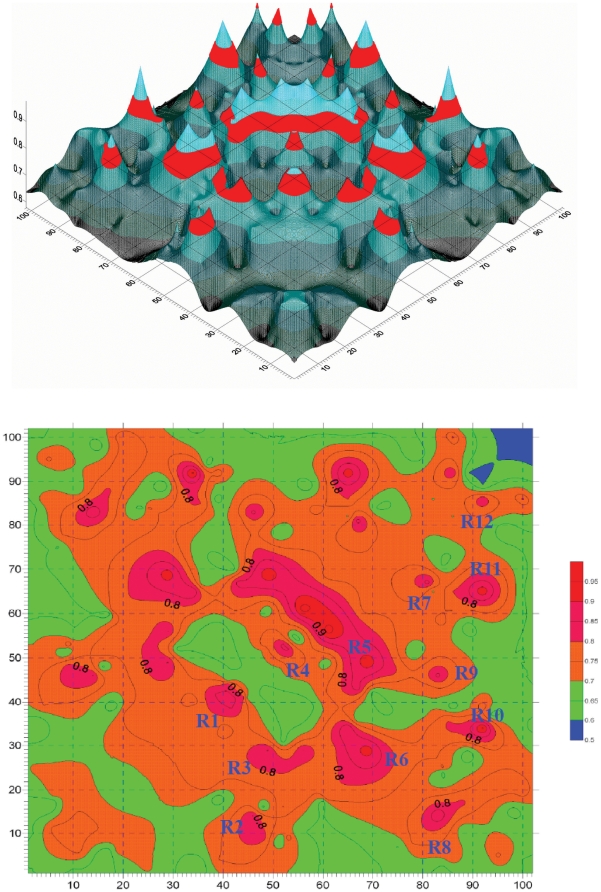
Graphical representation of position mutation correlation matrix R^(mut)^_L×L_ of PDZ. (**A**) The relief map of position mutation correlation matrix R^(mut)^_L×L_ of PDZ database. The red bands indicate the region with correlation coefficients between 0.80 to 0.85. The relief map of R^(mut)^_L×L_ is complementary to the relief map of position conservation correlation matrix R^(con)^_L×L_ (Fig. 2). (**B**) The contour map of position mutation correlation matrix R^(mut)^_L×L_ of PDZ database. For a clear view the matrix elements *r* ^(mut)^ *_i,j_* less than 0.5 are filtered, and the elements (*r* ^(mut)^ *_i,i_* = 1) on diagonal line are not shown. The position mutation correlation matrix R^(mut)^_L×L_ is symmetric to the diagonal line. The map is colored according to the values: the regions with value higher than 0.90 are colored in red, higher than 0.80 in pink, and higher than 0.70 in orange. In the map there are 12 regions (R1 to R12), where the correlation coefficients *r* ^(mut)^ *_i,j_* are higher than 0.80.

Then we perform the mutation position correlation analysis to the PDZ protein family. The position mutation correlation matrix R^(mut)^_L×L_ of PDZ is graphically shown in Fig. 3.

The matrix R^(mut)^_L×L_ is symmetric to the diagonal line. For a clear view the matrix elements *r* ^(mut)^ *_i,j_* less than 0.5 are filtered, and the elements on the diagonal line (*r* ^(mut)^ *_i,i_* = 1) are not shown. Fig. 3 A is the relief map of position mutation correlation matrix R^(mut)^_L×L_ of PDZ database. The red bands indicate the region with correlation coefficients from 0.80 to 0.85. Fig. 3 B is the contour map of position mutation correlation matrix R^(mut)^_L×L_. The map is colored in the same manner as in Fig. 2.

After careful observation and comparison we find partially complementary relationship between the two correlation matrices R^(con)^_L×L_ and R^(mut)^_L×L_: the peaks and valleys are located in alternate places in Fig. 2 A and Fig. 3 A. And in Fig. 3 B there are less areas in red color than in Fig. 2 B. Only 12 separated red regions (R1 to R12) with higher correlation coefficients (*r_i,j_*>0.80) are found in Fig. 3 B. It is easier to find useful information from the position mutation correlation matrix R^(mut)^_L×L_ than that from the position conservation correlation matrix R^(con)^_L×L_.

### Information from CMCA of PDZ

Several studies have highlighted the presence of interaction networks within single-domain proteins, which are crucial for allostery, stability, and folding [2], [17], [34], [35], [36]. Based on the statistical coupling analysis (SCA) [2] PDZ domains were proposed to contain energetically coupled positions between residues located in the binding site and elsewhere, forming a long-range interaction network.

**Figure 4:**
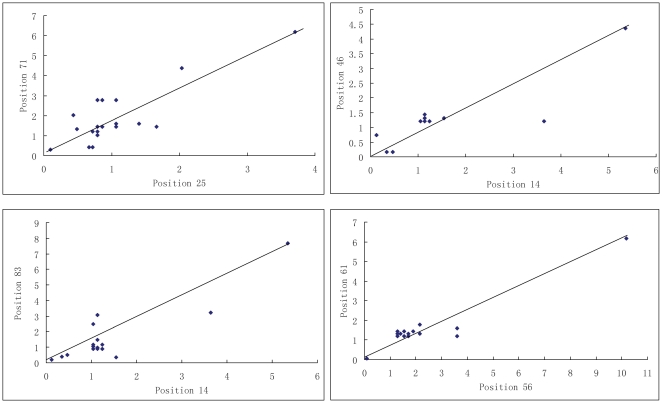
Amino acid mutation correlation relationships of 4 couple position pairs. The 4 position pairs have high mutation correlation coefficients (R_71−25_ = 0.8388, R_46−14_ = 0.8522, R_83−14_ = 0.892, and R_61−56_ = 0.9742), and the position mutation factors (*t_k,l_*) of 20 amino acid types at the couple position pairs show higher correlation relationship.

Table 1 lists 24 position pairs with higher correlation coefficients (*r* ^(mut)^ *_i,j_*>0.80) in the mutation correlation matrix R^(mut)^_L×L_, which distribute in 12 red regions in Fig. 3 B. In Table 1 the 24 position pairs are numbered according to the PDZ database [33]. The corresponding position numbers in the PDZ protein 1BE9 [25] are also listed in Table 1, which is a well investigated PDZ protein. Total 30 different positions are in the 24 position pairs. Among the 30 positions, 3 positions are gaps in the protein 1BE9. The correlations of four position pairs are shown in Fig. 4, which possess higher mutative correlation coefficients (R_71−25_ = 0.8388, R_46−14_ = 0.8522, R83−14 = 0.892, and R_61−56_ = 0.9742). The position 14 is highly correlated to both position 46 and position 83. Therefore, the three positions form a mutually mutative group (14, 46, 83) in the PDZ family.

**Table 1:** Mutation couple pairs with higher correlation coefficients in position mutation correlation matrix of PDZ database.

Mutation region^a^	Couple pair^b^		Correlation coefficient	1BE9 numbering	
	i	j	R_i,j_	i	j
R1	41	44	0.826708	36	39
	42	38^c^	0.847115	37	–c
R2	46	14	0.852198	41	16
	48	9	0.827886	43	11
R3	49	29	0.852867	44	30
	56	29	0.850957	51	30
R4	53	52	0.857384	48	47
R5	61	56	0.974221	56	51
	67	43	0.868664	62	38
	69	49	0.934357	64	44
	69	56	0.813315	64	51
	74	47	0.820122	69	42
R6	64	26	0.813597	59	27
	65	34^c^	0.896786	60	–c
	69	29	0.926572	64	30
	71	25	0.838772	66	26
R7	81	67	0.871094	76	62
R8	83	14	0.892220	78	16
R9	83	46	0.865799	78	41
R10	86	34^c^	0.809981	81	–c
	92	34^c^	0.959904	86	–c
R11	86	65	0.800912	81	60
	92	65	0.934498	86	60
R12	92	86	0.844610	86	81

a The regions (R1 to R12) are shown in **Fig. 3** (the contour map of position mutation correlation matrix).

b The position numbers are from PDZ database of ref [33].

c The positions indicated by superscript ‘c’ are the gaps in the 1BE9, which are inserted in multiple alignment.

The structure alignment of four PDZ proteins (2QKT, 2F5Y, 1G9O, and 1BE9) and results of position mutation correlation analysis are shown Fig. 1. The 27 residues, shown in stick render in Fig. 1 A, are located at the positions having higher mutative correlation coefficients (see Table 1). The surface of α2-β2 groove of 1BE9 and the peptide ligand is shown in Fig. 1 B. Blue is for hydrophilic surface and green is for hydrophobic surface. Fig. 1 C shows the residues of PDZ proteins 1BE9 and 2QKT at the positions controlling the ligand affinity. The size of Tyr79 and Leu81 of 2QKT (blue) are much larger than the corresponding residues Ala76 and Ala78 of 1BE9 (green). The PDZ protein 2QKT has a disulfide bond between Cys37 and Cys78 shown in Fig. 1 D, which makes it different from other PDZ proteins [26], [37]. The position 37 and 78 are easily mutative positions based on CMCA results in Table 1. Fig. 1 E shows the sequence alignment of four PDZ proteins (2QKT, 2F5Y, 1G9O, and 1BE9). The residues, at the positions having higher position mutation correlation coefficients (see Table 1), are indicated by green frames.

The 27 mutative positions with higher mutation correlation coefficients distribute in two α-helices, all 6 β-strands, and some loops, including the easily mutative positions and some very conservative positions [33]. In Fig. 1 E among the 27 positions there are two larger sectors, each of them consists of four adjacent positions: 36–39 (in β3) and 41–44. Some interesting findings are summarized as follows.

### Controlling positions for ligand affinity

The groove between α2 helix and β2 strand is the binding location for ligand peptide [2], [38] and the residues at these positions are highly conservative. However, mutations at these highly conservative positions may have more important significance to biological functions. Four easily mutative positions are found in the α2-β2 groove: Asn26 and Ile27 in β2, Ala76 and Ala78 in α2 (1BE9 numbering), which determine the ligand binding affinity and control the peptide shape and specificity. In Fig. 1 C the small residues Ala76 and Ala78 (in green) of 1BE9 are replaced by Tyr79 and Leu81 (in blue) of 2QKT. The size of Tyr79 and Leu81 of 2QKT are much larger than the Ala76 and Ala78 of 1BE9. Therefore 1BE9 and 2QKT must have very different preferences of peptide ligand.

The biological relevance of long-range allosteric effects in PDZ domains has attracted considerable attention [7], [39], [40]. The PDZ domain of the cell polarity protein Par6 was shown to be allosterically regulated by its adjacent Crib domain in response to binding of CdC42 [39]. Structural analysis showed the β1-α1 interface of the Par6 PDZ domain to be in direct contact with the Crib domain and responsible for transmission to the structurally distinct peptide binding pocket [41]. The results of CMCA fully support above observations. Two easily mutative positions (Ala47 and Asp48, in 1BE9 numbering) are found in α1 helix, and two positions Pro11 and Ile16 (in 1BE9 numbering) are found in β1 strand. These easily mutative positions are connected through peptide ligand and define an allosteric mechanism for regulating binding affinity at the α2-β2 groove through molecular interactions at a distant surface site on the α1 helix [39].

### Disulfide bond in INAD PDZ5

In the alignment of four PDZ proteins in Fig. 1 E the 2QKT [37] is an INAD PDZ [42] domain and belongs to type 5 PDZ. The INAD PDZ domain (PDZ5) exists in a redox-dependent equilibrium [43], [44] between two conformations—a reduced form that is similar to the structure of other PDZ domains, and an oxidized form. In INAD PDZ an intramolecular disulfide bond covalently links a pair of buried cysteine residues located underneath the floor of the ligand-binding pocket [26], [37]. In 2QKT the disulfide bond is formed between Cys37 in β3 and Cys78 in α2 (in Fig. 1 E numbering). The positions of Cys37 and Cys78 are corresponding to the positions of residues Ile36 and Ala75 of 1BE9, respectively. The position 36 (in 1BE9 numbering) is an easily mutative position according to results of CMCA calculations (see Table 1), and the position 75 (in 1BE9 numbering) is adjacent to the easily mutative position 76 (see Table 1) falling into the mutative region R7 (see Fig. 3 B). The strong intramolecular disulfide bond connects the β3 strand with the α2 helix, suggesting that this interaction may be responsible for the equilibrium between the reduced conformation and the oxidized conformation in INAD PDZ5.

### Distantly allosteric network in PDZ proteins

The functional coupling of two positions, even if distantly positioned in the structure, could mutually constrain evolution at the two positions, and these should be represented in the statistical coupling of the underlying amino acid distributions [2], [4], [23]. In some cases the functional coupling is not limited only between two positions, but could be among several distant positions, which form a mutually evolutionary network.

Long-range allosteric effects that cause the preference change of ligand peptide in the PDZ binding groove happen in several distant positions. The results of CMCA study reveal the mutually multi coupling positions in the PDZ family. Table 1 lists the couple pairs of easily mutative positions. Actually, these positions can be reorganized into three groups according to the mutual couple pairs (in 1BE9 numbering): (30, 44, 51, 56, 64), (16, 41, 78), and (60, 81, 86). In the group 2 the three positions are at distantly separated β1 (position 16), β3 (position 41), and α2 (position 78) that may form a mutually mutative network and may affect the binding sites in α2-β2 groove. These findings could provide an explanation to distant allosteric interaction network in PDZ proteins.

## Discussion

Conservation and mutation are two opposite aspects of functional evolution of protein family. In the studies for protein evolutionary family the conversation statistical analysis can provide useful information, and several successful tools are developed based on the position conversations, such as SCA (statistical coupling analysis) [2], [15] and MI (mutual information) [12], [13]. In this study we prove that correlation analysis based on position mutations of amino acids also can reveal very useful information for study of functional evolution of protein family. The position mutations are equally important to the position conservations for study of functional evolution of protein family.

In the conservation-based statistical methods the “phylogenetic relationship” in a protein family causes the “coherent correlation” of all positions [33], which is raised by sharing common ancestry. Great efforts have been made to solve the “coherent correlation” problem [12], [13]. In this study the foundational equation is the amino acid position mutative function (Eq.7), based on which the amino acid position mutation matrix T_M×L_ is constructed, and the CMCA approach is developed. Unlike the conservation-based methods, the “coherent correlation” problem may be avoided in the mutation-based method. The theoretical implications of Eq.7 and CMCA approach are summarized as follows. (i) The actual mutations at specific directions are caused by the functional constraints in the protein evolutionary family. The directional mutations at some key positions control the functional evolution of the protein family. (ii) The functional coupling of two or more positions, even if distantly positioned in the structure, mutually constrains mutations at these positions, which form a communicative and evolutionary network in the protein family.

The computation results of CMCA application to the PDZ protein database show that generalizing the principle of mutations to account for correlations between positions reveals a novel structural organization for PDZ proteins that is distinct from traditional structural descriptions and the SCA approach. The CMCA approach and the SCA approach describe the distant allostery and mutative network in protein evolutionary family from different aspects (mutations and conservations), therefore both methods can provide useful information complementally.

Because the conservation-mutation correlation analysis is based on the correlation analysis of amino acid mutations, the CMCA approach may find applications in rational protein design and enzyme engineering by means of artificial residue mutations, and provide suggestion to improve the bioactivities and physicochemical properties of enzymes.

## Materials and Methods

Evolutionarily related proteins have similar sequences and naturally occurring homologous proteins have similar protein structures. It has been shown that three-dimensional protein structure is evolutionarily more conserved than expected due to sequence conservation [45], [46]. However, the evolution of protein family mainly depends on the mutations happening on the key positions in the 3D structures. Statistical analysis for a protein evolutionary family starts from a multiple 3D structural alignment (MSA) of a homologous protein group.

### Multiple structure alignment of protein family

In this study, the multiple structure alignment procedure is used. Chains that possess coordinates for all their alpha carbons can be realigned taking into account their structure. From an initial estimate of the alignment, a new similarity matrix is generated using the relative alpha carbon coordinates that result from a multi-body superposition. This matrix is used to realign just these alpha carbon populated chains. This procedure is then repeated until the Root Mean Square Distance (RMSD) of the superposition fails to improve. The multiple structural alignment of a protein family has to reveal the structural features: all key functional residues are aligned in same sequence columns, and all key secondary structures (α-helices, β-sheets, and loops) are positioned in the same sectors.

After multiple sequence alignment the protein family is represented by a three dimensional primer data matrix X^(0)^_N×M×L_. N is the number of protein structures in the database, M is the types of amino acids (M = 21, including 20 natural amino acids and the gap, which are inserted during the multiple alignment), and L is the length of amino acid sequences (including gaps). The database matrix X^(0)^_N×M×L_ is a binary matrix, in which the element x^(0)^ *_i,k,l_* of sequence *i* at position *l* is 1 when the amino acid is *a_k_*, otherwise, it is 0,
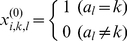
(1)


### Amino acid position frequency matrix

From the primer data matrix X^(0)^_N×M×L_ the primer amino acid position frequency matrix F^(0)^_M×L_ is constructed as follows,
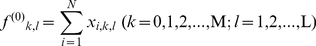
(2)The value of *f* ^(0)^ *_k,l_* is an integer in region [0,N], equals the times of amino acid *a_k_* appearing at position *l* in all N protein sequences. The higher value of *f* ^(0)^ *_k,l_* means the higher frequency of amino acid *a_k_* at position *l*. In this study the gaps are treated as a special amino acid type numbered by 0, and the 20 natural amino acids are numbered from 1 to 20. The summation of *f* ^(0)^ *_k,l_* from *k* = 0 to M is N. The F^(0)^_M×L_ is integer frequency matrix of amino acids. It can be transformed to decimal frequency after dividing by N.

### Reducing unnecessary gaps

The position correlation analysis is complicated by the presence of alignment gaps, commonly called indels, indicating the structural region present in some proteins but not in others. The gaps (space positions) in the primer data matrix X^(0)^_N×M×L_ may interfere with the results of statistical analysis badly. Before performing the correlation analysis we have to reduce the unnecessary gaps. To do so, the total amino acid frequencies of 20 natural amino acids at each position *l* are computed as follows.
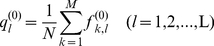
(3)In Eq.3 the index *k* for amino acid types is from 1 to M = 20, not including the gap. If the total amino acid position frequencies of 20 natural amino acids *q* ^(0)^ *_l_* is less than 20%, the position *l* is deleted from the primer sequences. Because it means that at the position *l* more than 80% ‘amino acids’ are gaps, and this position is less important for the biological function of the protein family. After unnecessary gaps are deleted, we get the reduced data matrix X_N×M×L_ and amino acid position frequency matrix F_M×L_, in which the sequence length L is smaller than in the primer data matrix. For simplicity, we still use L for the reduced sequence length.

### Position conservation correlation matrix

The position conservation correlation matrix can be derived from the reduced position frequency matrix F_M×L_. Because the conservation is directly correlated to the amino acid position frequency, the higher frequency *f_k,l_* of amino acid *k* at position *l*, the more conservation of amino acid *k* at this position. For position conservation correlation analysis the position frequency covariance matrix C^(con)^_L×L_ is constructed firstly from the reduced position frequency matrix F_M×L_,
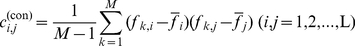
(4)where 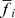 and 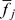 are the average frequencies at position *i* and *j*, respectively,
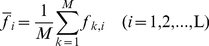
(5)Hereby we get the position conservation correlation matrix R^(con)^_L×L_ from the position covariance matrix C^(con)^_L×L_ as follows.
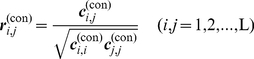
(6)where the superscript ‘con’ indicates the ‘conservation’, and ***r*** ^(con)^ *_i,j_* is the position conservation correlation coefficient between position *i* and *j*.

### Position mutation correlation matrix

Before computing the amino acid position mutation correlation matrix, we have to build the amino acid position mutative equation, which measures the mutation of amino acid *k* at position *l* in protein family. For this purpose the amino acid types *n_l_* (including gap) at each position *l* in the protein family is very useful, which describes the diversification of amino acids at position *l*. The larger value of *n_l_*, the more mutations at position *l*. The amino acid position mutation matrix T_M×L_ is constructed as follows.
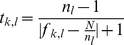
(7)The value *t_k_*_,*l*_ is the measurement of mutation of amino acid *k* at position *l* in protein family, which is directly proportional to the amino acid types *n_l_* at position *l* and inversely proportional to the integer frequency *f_k_*_,*l*_ of amino acid *k* at position *l*. The term N/*n_l_* in denominator is the average frequency at position *l*. The ‘1’ in denominator is added to avoid the infinite value when the frequency of amino acid *k* at position *l* equals to the average frequency *f_k_*_,*l*_ = N/*n_l_*. The ‘1’ in numerator makes the value of *t_k_*_,*l*_ is 0 for all amino acid types when *n_l_* is 1 (no mutation at position *l*).

**Figure 5:**
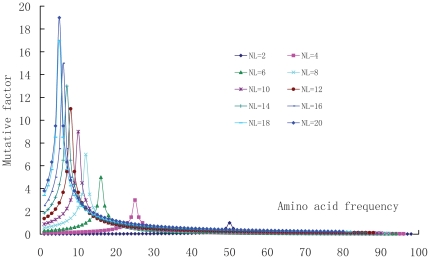
The mutative factor function (**Eq.7**) used in conservation-mutation correlation analysis (CMCA). The curves are computed according to Eq.7. The horizontal coordinates are the integer position frequencies *f_k,l_* of amino acids in protein database, and the vertical coordinates are the mutative factors. The *n_l_* is the amino acid types at position *l*. In calculations the protein sample number N is 100. When the frequency of amino acid *k* takes the average value *f_k,l_* = N/*n_l_*, all curves have the maximum values, and when the amino acid types at position *l* has the largest value (*n_l_* = 20), the mutative factor gets the largest value 19.

Fig. 5 shows the curve shapes of amino acid position mutative equation (Eq.7). When the frequency of amino acid *k* takes the average value at position *l* (*f_k_*_,*l*_ = N/*n_l_*) all curves have the maximum values, and when the amino acid type at position *l* has the largest value (*n_l_* = 20), the mutative factor gets the largest value.

**Figure 6:**
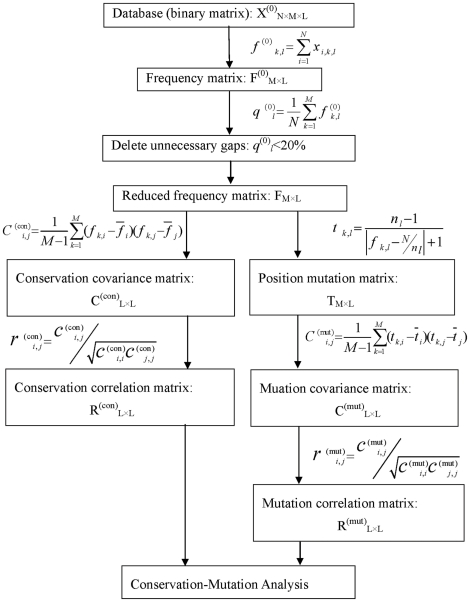
The flowchart of conservation-mutation correlation analysis (CMCA). The binary matrix X^(0)^_N×M×L_ is the primer database of protein evolutionary family, and F^(0)^_M×L_ is the primer amino acid position frequency matrix. Integer N is the number of protein samples, M = 21 is the types of amino acids (including the gaps), and L is the length of protein sequences. After the unnecessary gaps are deleted, the above two matrices are denoted as X_N×M×L_ and F_M×L_. From the frequency matrix F_M×L_ the amino acid position conservation correlation matrix R^(con)^_L×L_ is constructed, and from the amino acid position mutation matrix T_M×L_ the amino acid position mutation correlation matrix R^(mut)^_L×L_ is constructed.

Following the same procedure described in sector 2.4, we can construct the position mutation covariance matrix C^(mut)^_L×L_ from amino acid position mutation matrix T_M×L_,
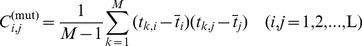
(8)where 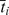 and 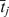 are the average mutations at position *i* and *j*, respectively,
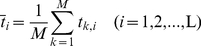
(9)Hereby we get the position mutation correlation matrix R^(mut)^_L×L_ from the position mutation covariance matrix C^(mut)^_L×L_ as follows.
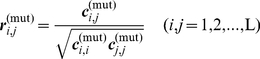
(10)where ***r*** ^(mut)^ *_i,j_* is the position mutation correlation coefficient between position *i* and *j*. The computational procedure is graphically illustrated in Fig. 6, the flowchart of conservation-mutation correlation analysis (CMCA).

## References

[1] Galtier N, Dutheil J (2007). Coevolution within and between Genes.. Gene and Protein Evolution.

[2] Lockless SW, Ranganathan R (1999). Evolutionarily conserved pathways of energetic connectivity in protein families.. Science.

[3] Hatley ME, Lockless SW, Gibson SK, Gilman AG, Ranganathan R (2003). Allosteric determinants in guanine nucleotide-binding proteins.. Proc Natl Acad Sci U S A.

[4] Neher E (1994). How frequent are correlated changes in families of protein sequences?. Proc Natl Acad Sci U S A.

[5] Gobel U, Sander C, Schneider R, Valencia A (1994). Correlated mutations and residue contacts in proteins.. Proteins.

[6] Shulman AI, Larson C, Mangelsdorf DJ, Ranganathan R (2004). Structural determinants of allosteric ligand activation in RXR heterodimers.. Cell.

[7] Swain JF, Gierasch LM (2006). The changing landscape of protein allostery.. Curr Opin Struct Biol.

[8] Benkovic SJ, Hammes-Schiffer S (2003). A perspective on enzyme catalysis.. Science.

[9] Hammes-Schiffer S, Benkovic SJ (2006). Relating protein motion to catalysis.. Annu Rev Biochem.

[10] Henzler-Wildman K, Kern D (2007). Dynamic personalities of proteins.. Nature.

[11] Luque I, Leavitt SA, Freire E (2002). The linkage between protein folding and functional cooperativity: two sides of the same coin?. Annu Rev Biophys Biomol Struct.

[12] Dunn SD, Wahl LM, Gloor GB (2008). Mutual information without the influence of phylogeny or entropy dramatically improves residue contact prediction.. Bioinformatics.

[13] Martin LC, Gloor GB, Dunn SD, Wahl LM (2005). Using information theory to search for co-evolving residues in proteins.. Bioinformatics.

[14] Dutheil J, Galtier N (2007). Detecting groups of coevolving positions in a molecule: a clustering approach.. BMC Evol Biol.

[15] Lichtarge O, Bourne HR, Cohen FE (1996). An evolutionary trace method defines binding surfaces common to protein families.. J Mol Biol.

[16] Cover TM, Thomas JA (2006). Elements of information theory.

[17] Socolich M, Lockless SW, Russ WP, Lee H, Gardner KH (2005). Evolutionary information for specifying a protein fold.. Nature.

[18] Looger LL, Dwyer MA, Smith JJ, Hellinga HW (2003). Computational design of receptor and sensor proteins with novel functions.. Nature.

[19] Carmena A, Speicher S, Baylies M (2006). The PDZ protein Canoe/AF-6 links Ras-MAPK, Notch and Wingless/Wnt signaling pathways by directly interacting with Ras, Notch and Dishevelled.. PLoS One.

[20] Boxus M, Twizere JC, Legros S, Dewulf JF, Kettmann R (2008). The HTLV-1 Tax interactome.. Retrovirology.

[21] Ponting CP (1997). Evidence for PDZ domains in bacteria, yeast, and plants.. Protein Sci.

[22] Walsh NP, Alba BM, Bose B, Gross CA, Sauer RT (2003). OMP peptide signals initiate the envelope-stress response by activating DegS protease via relief of inhibition mediated by its PDZ domain.. Cell.

[23] Ozkan E, Yu H, Deisenhofer J (2005). Mechanistic insight into the allosteric activation of a ubiquitin-conjugating enzyme by RING-type ubiquitin ligases.. Proc Natl Acad Sci U S A.

[24] Te Velthuis AJ, Isogai T, Gerrits L, Bagowski CP (2007). Insights into the molecular evolution of the PDZ/LIM family and identification of a novel conserved protein motif.. PLoS One.

[25] Doyle DA, Lee A, Lewis J, Kim E, Sheng M (1996). Crystal structures of a complexed and peptide-free membrane protein-binding domain: molecular basis of peptide recognition by PDZ.. Cell.

[26] Hung AY, Sheng M (2002). PDZ domains: structural modules for protein complex assembly.. J Biol Chem.

[27] Kurakin A, Swistowski A, Wu SC, Bredesen DE (2007). The PDZ domain as a complex adaptive system.. PLoS One.

[28] Wilken C, Kitzing K, Kurzbauer R, Ehrmann M, Clausen T (2004). Crystal structure of the DegS stress sensor: How a PDZ domain recognizes misfolded protein and activates a protease.. Cell.

[29] Daniels DL, Cohen AR, Anderson JM, Brunger AT (1998). Crystal structure of the hCASK PDZ domain reveals the structural basis of class II PDZ domain target recognition.. Nat Struct Biol.

[30] Morais Cabral JH, Petosa C, Sutcliffe MJ, Raza S, Byron O (1996). Crystal structure of a PDZ domain.. Nature.

[31] Ranganathan R, Ross EM (1997). PDZ domain proteins: scaffolds for signaling complexes.. Curr Biol.

[32] Bauler TJ, Hendriks WJ, King PD (2008). The FERM and PDZ domain-containing protein tyrosine phosphatases, PTPN4 and PTPN3, are both dispensable for T cell receptor signal transduction.. PLoS One.

[33] Halabi N, Rivoire O, Leibler S, Ranganathan R (2009). Protein sectors: evolutionary units of three-dimensional structure.. Cell.

[34] Macias MJ, Gervais V, Civera C, Oschkinat H (2000). Structural analysis of WW domains and design of a WW prototype.. Nat Struct Biol.

[35] Suel GM, Lockless SW, Wall MA, Ranganathan R (2003). Evolutionarily conserved networks of residues mediate allosteric communication in proteins.. Nat Struct Biol.

[36] Di Cera E (2004). Thrombin: a paradigm for enzymes allosterically activated by monovalent cations.. C R Biol.

[37] Mishra P, Socolich M, Wall MA, Graves J, Wang Z (2007). Dynamic scaffolding in a G protein-coupled signaling system.. Cell.

[38] Coleman SK, Cai C, Kalkkinen N, Korpi ER, Keinanen K (2010). Analysis of the potential role of GluA4 carboxyl-terminus in PDZ interactions.. PLoS One.

[39] Peterson FC, Penkert RR, Volkman BF, Prehoda KE (2004). Cdc42 regulates the Par-6 PDZ domain through an allosteric CRIB-PDZ transition.. Mol Cell.

[40] Schlieker C, Mogk A, Bukau B (2004). A PDZ switch for a cellular stress response.. Cell.

[41] Atwood SX, Chabu C, Penkert RR, Doe CQ, Prehoda KE (2007). Cdc42 acts downstream of Bazooka to regulate neuroblast polarity through Par-6 aPKC.. J Cell Sci.

[42] Morrison DK, Davis RJ (2003). Regulation of MAP kinase signaling modules by scaffold proteins in mammals.. Annu Rev Cell Dev Biol.

[43] Hanson GT, Aggeler R, Oglesbee D, Cannon M, Capaldi RA (2004). Investigating mitochondrial redox potential with redox-sensitive green fluorescent protein indicators.. J Biol Chem.

[44] Ostergaard H, Tachibana C, Winther JR (2004). Monitoring disulfide bond formation in the eukaryotic cytosol.. J Cell Biol.

[45] Chothia C, Lesk AM (1986). The relation between the divergence of sequence and structure in proteins.. EMBO J.

[46] Kaczanowski S, Zielenkiewicz P (2010). Why similar protein sequences encode similar three-dimensional structures?.. Theor Chem Acc.

